# Genomic analysis of virulence factors of nosocomial MDR *A. baumannii* from a tertiary care hospital

**DOI:** 10.1128/spectrum.00707-25

**Published:** 2025-11-11

**Authors:** Asmabanu Shaikh, Anant Marathe, Bhavita Prajapati

**Affiliations:** 1 Department of Microbiology, Parul Institute of Medical Sciences & Research, Faculty of Medicine, Parul University 569346 , Vadodara, Gujarat, India; Tainan Hospital Ministry of Health and Welfare, Tainan, Taiwan

**Keywords:** virulence factors, MDR *A. baumannii*, ventilator-associated pneumonia, whole-genome sequencing

## Abstract

*Acinetobacter baumannii* has evolved over the years into predominant nosocomial multidrug-resistant (MDR) pathogen, especially associated with ventilator-associated pneumonias (VAPs). About one-third of VAPs can cause bloodstream infections. MDR *A. baumannii* causing VAP is associated with significant longer hospital stays and mortality. We carried out the genomic analysis of MDR *A. baumannii* by whole-genome sequencing (WGS) to study the various virulence factors involved in biofilm formation and pathogenic mechanisms. A total of 44 MDR *A. baumannii* isolates recovered from respiratory and blood specimens of patients on mechanical ventilators are included in the study. All isolates were identified by Vitek2 and matrix-assisted laser desorption/ionization–time of flight mass spectrometry and confirmed by WGS. Different virulence factor (VF) genes identified by WGS were analyzed. The most common VF genes found in all isolates of *A. baumannii* were *ompA* (97.7%) and *lps-o* gene (100%) related to adherence, *adeF* (100%), *adeG* (95.5%), and *adeH* (97.7%) (AdeFGH efflux pump-related VF genes) and *pgaA* and *pgaD* (100%), *pgaB* and *pgaC* (97.7%) related to biofilm formation, *plc* and *plcD* (100%) related to enzyme and immune evasion related capsular gene (100%) and *lpsB, lpxA, lpxC, lpxD, lpxL*, and *lpxM* found in all isolates respectively. This study is amongst a few such studies done using WGS in India. These findings have improved our understanding of the various pathogenic mechanisms underlying *A. baumannii*. This study will also give some insight into how hindering the pathways of virulence mechanism can be used to develop treatment strategies for MDR *A. baumannii*.

**IMPORTANCE:**

*Acinetobacter baumannii* is a significant cause of mortality in nosocomial infections, particularly ventilator-associated pneumonia (VAP). The evolution of *A. baumannii* from an avirulent organism to a multidrug-resistant (MDR) nosocomial pathogen is very interesting. The acquisition of multiple drug-resistant genes is also accompanied by the acquisition of many virulence genes. Information regarding genomic studies of different virulence factors in such MDR strains, prevalent in this geographical region, were not performed in the past. The strains of *A. baumannii* may vary depending on the geographical location and use of antibiotics in those hospital setups. Control of *A. baumannii* nosocomial infections requires taking strict hospital infection control measures. We trust that this would suggest some new insights into the control of *A. baumannii* in our intensive care units to reduce the burden of such a dreadful nosocomial pathogen.

## INTRODUCTION

*Acinetobacter baumannii* is an opportunistic, oxidase-negative, gram-negative coccobacillus. In addition to ventilator-associated pneumonia (VAP), this pathogen can cause a range of serious infections, , bloodstream infections (BSIs), intra-abdominal infections, meningitis, and post-surgical site infections (1). Carbapenem-resistant *A. baumannii* (CRAB) is now categorized as a top-priority pathogen by WHO (1).

From early 1990 to 2024, *A. baumannii* has significantly transformed from a highly susceptible pathogen to a multidrug-resistant (MDR) pathogen (2). The incidence rate of VAPs ranged from 2.13 to 116 cases per 1,000 ventilator days, showing significant variation between countries (3). MDR/extensively drug-resistant (XDR) strains of *A. baumannii* are responsible for up to 20% of nosocomial infections worldwide, with mortality rates reported as high as 84.3% in cases of VAP in ICU patients (4).

*A. baumannii* is a pathogen that can adapt quickly to challenging environmental conditions in the hospitals owing to its genomic plasticity (5). This characteristic of *A. baumannii* helps in developing rapid antibiotic resistance and survival in the hospital environment. The various virulence factors (VFs) responsible for the persistence of *A. baumannii* comprise capsular polysaccharides, biofilm-associated proteins, capsular lipopolysaccharides (LPSs), outer membrane vesicles, and secretion systems.

VFs in *A. baumannii* have many genes encoding various VFs. OmpA assists in adherence to the host cell surface and induces apoptosis by activating caspases (5, 6). Fimbriae, efflux pump, and biofilm-associated proteins are mainly involved in biofilm formation (5, 7). Pili promote adherence and biofilm formation (5). LPSs are involved in triggering the host’s immune response and evasion of immune response (8). Outer membrane vesicles are involved in antibiotic resistance (5). Capsular polysaccharides help in prolonged bacterial survival in tissues and help in biofilm formation (9). Phospholipase enables *A. baumannii* cells to cause lysis of the host cells by cleaving the phospholipids present in the cell membrane of the host cells, making tissue evasion easy for them (10). BfmRS participates in cell adhesion, biofilm formation, resistance to complement-associated killing, and resistance to antibiotics (10, 11). Type II secretion system is essentially involved in the secretion of enzymes like lipases and proteases, while type VI secretion system has a competitive role in the acquisition of antibiotic resistance. These factors contribute to antibiotic resistance and immune evasion (12).

Over the last two decades, although there have been some study reports about the antibiotic resistance pattern of *A. baumannii* from various geographical locations but the studies focusing on the VFs are scarce.

The present study focuses on the various VFs of *A. baumannii* and their role in pathogenicity and clinical outcome of such infections. We also tried to find the relationship between the host factors like comorbidities associated with the infection. The VF genes of *A. baumannii* were studied by whole-genome sequencing (WGS).

## MATERIALS AND METHODS

### Study design

The study was conducted after ethical approval at a tertiary care hospital in Baroda, Gujarat, India from April 2023 to January 2024. The Parul Sevashram Hospital is a tertiary care facility with the capacity to accommodate approximately 600 patients in the ICU. During the study period, a total of 1,467 patients were admitted to the ICUs, out of which 695 needed ventilator support.

Out of 101 *A*. *baumannii* (MDR/XDR) isolates, WGS was performed on first 44 isolates (21 BSI and 23 respiratory specimens mainly endotracheal secretion and tracheal secretion) recovered from patients on mechanical ventilators included in the study.

### Identification of isolates by VITEK2

Identification by VITEK 2 compact system: All clinical isolates were identified using the gram-negative identification (GN ID) card, with an expected identification rate of over 90%.

### Identification by matrix-assisted laser desorption/ionization–time of flight mass spectrometry (MALDI TOF MS) (Bruker MALDI Biotyper, version 4.1.100)

A small sample from a freshly grown bacterial colony was applied to the MALDI target plate and immediately overlaid with 1 µL of CHCA (α-cyano-4-hydroxycinnamic acid) matrix solution (13). After drying, the prepared target plate was inserted into the mass spectrometer for analysis. Bacterial identification was performed using the Bruker MALDI Biotyper software (version 4.1.100).

### Molecular methods

The DNA of bacteria was extracted manually using the CTAB method (14). To ensure its quality, gel electrophoresis was performed. Once the DNA passed the quality check, it was utilized for the preparation of DNA libraries, following the manufacturer,s instructions (Illumina Nextera XT). The quality of these libraries was then assessed by the QIAXEL machine. The DNA libraries were sequenced using the Illumina NextSeq kit.

High-throughput sequencing was performed on the Illumina NovaSeq 6000 platform. Raw reads were subjected to quality control using FastQC on a Linux-based system.

### Assembly and annotations of the genome

The Bacterial and Viral Bioinformatics Resource Center version 3.35.5 was used for genome annotation and assembly (15). The isolates were identified using PubMLST software (16).

### Multilocus sequence typing (MLST) typing

MLST of *A. baumannii* genomes was performed using the online MLST v2.0 tool available at the Center for Genomic Epidemiology (17). The Pasteur scheme was employed to sequence seven core genes, including *recA*, *rplB*, *cpn60*, *gltA*, *fusA*, *pyrG*, and r*poB*, which are essential for identifying the genetic diversity of *A. baumannii* isolates (17).

### Prediction of VF genes

The VF analyzer software utilizes a comprehensive approach to identify VFs in bacterial genomes. It constructs orthologous groups by comparing the query genome to reference genomes from the Virulence Factor Database (VFDB), which comprises multiple strains of *A. baumannii*, including *A. baumannii* 1656-2, AB0057, AB307-0294, ACICU, ATCC 17978, AYE, BJAB07104, BJAB0715, BJAB0868, D1279779, MDR-TJ, MDR-ZJ06, SDF, TCDC-AB0715, and TYTH-1. This method minimizes false positives resulting from paralogous genes (15). The software conducts extensive sequence similarity searches within the VFDB to detect potential VFs that may be specific to certain strains or atypical in nature. Furthermore, it employs a context-based refinement process for VFs encoded by gene clusters, ensuring high accuracy and reliability in its results without requiring manual intervention (15).

Performed *t*-test and Mann-Whitney test to find *P* value by using the software Jamovi 2.6.44.0 version.

## RESULTS

### Identification of isolates

Forty-four out of 101 isolates of *A. baumannii* (MDR/XDR) were included in the study. Out of 44 isolates, 21 (48%) were isolated from blood, 18 (41%) from endotracheal secretion, and 5 (11%) from tracheal secretion, respectively (Table 1).

**TABLE 1:** Type of specimen from which *A. baumannii* isolated

Type of specimen	Number of isolates
Blood	21 (48%)
Endotracheal secretion	18 (41%)
Tracheal secretion	5 (11%)
Total	44

The species identification of isolates was done by VITEK 2 as well as MALDI TOF MS. The concordance in species identification by two methods was found to be 100%. Identification of all the isolates was further confirmed by WGS.

Table 2 presents the genomic characteristics of *A. baumannii* isolates, including genome length, number of contigs, *N*_50_, and GC content.

**TABLE 2:** Details of sequence data of *A. baumannii* isolates

Sample no.	NCBI no.	Genome length	No. of contigs	*N*_50_	GC content
LNF3	JBMSWK000000000	3,881,451	64	144,238	38.80%
LNF20	JBNVTQ000000000	3,923,101	84	144,238	38.80%
AB 22	JBODTA000000000	3,934,032	86	139,998	39%
AB 23	JBODSZ000000000	3,943,009	72	170,259	39%
AB 41	JBODSY000000000	3,938,232	76	139,996	39%
AB 48	JBODSX000000000	3,943,108	72	170,259	39%
AB 54	JBODSW000000000	3,949,075	115	99,753	39%
AB 55	JBODSV000000000	3,847,141	118	115,329	39%
AB 57	JBODSU000000000	3,870,767	104	94,626	38.90%
AB 61	JBODST000000000	3,871,543	95	94,626	38.90%
AB 62	JBODSS000000000	3,855,252	97	94,625	38.90%
AB 63	JBODSR000000000	3,868,897	109	94,444	38.90%
AB 64	JBODSQ000000000	4,046,469	100	139,973	39.40%
AB 66	JBODSP000000000	3,872,662	97	94,444	38.90%
AB 69	JBODSO000000000	3,871,396	102	94,626	38.90%
AB 70	JBODSN000000000	3,943,736	115	91,806	38.8
AB 134	JBODSM000000000	3,969,851	103	129,125	38.90%
AB 139	JBODSL000000000	3,960,233	163	93,882	39%
AB 143	JBODSK000000000	4,062,393	83	169,529	38.90%
AB 152	JBODSJ000000000	4,228,056	308	57,205	39%
AB 181	JBODSI000000000	3,866,522	116	94,445	38.90%
AB 182	JBODSH000000000	3,860,122	120	94,445	38.90%
AB 183	JBODKO000000000	4,017,853	106	147,032	39%
AB 184	JBODKN000000000	3,982,099	110	147,031	39%
AB 190	JBODKM000000000	4,162,420	102	118,466	39%
AB 191	JBODKL000000000	3,868,033	103	88,666	38.90%
AB 197	JBODKK000000000	3,865,146	118	9,445	38.90%
AB 204	JBODKJ000000000	3,971,836	111	133,449	39%
AB 206	JBODKH000000000	3,474,295	1,057	4,204	39%
AB 212	JBODKG000000000	4,040,708	85	139,997	39%
AB 214	JBODKF000000000	4,042,735	82	174,349	39%
AB 216	JBODKE000000000	3,839,658	67	142,284	39%
AB 217	JBODKD000000000	3,834,946	117	83,981	39.20%
AB 218	JBODKC000000000	3,923,354	126	86,211	39%
AB 245	JBODKB000000000	3,944,017	70	176,648	39%
AB 246	JBODKA000000000	3,868,577	114	9,188	38.90%
AB 65.1	JBODJJ000000000	4,081,586	116	125,893	39%
AB 12.1	JBODJN000000000	3,936,011	114	114,280	38.80%
AB 42.1	JBODJM000000000	4,081,580	117	130,862	39.30%
AB 56.1	JBODJL000000000	4,074,239	102	133,449	39.10%
AB 61.1	JBODJK000000000	3,908,350	110	88,666	39%
AB 198.1	JBODJI000000000	3,904,295	190	71,413	38.90%
AB 207.1	JBODJH000000000	3,872,067	305	26,627	38.90%
AB 237.1	JBODJG000000000	4,052,633	114	139,997	39%

### Sequence type (ST)

**Fig 1:**
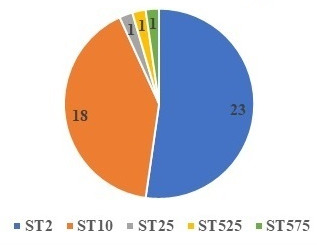
MLST type.

A total of 44 MDR *A. baumannii* isolates were characterized by MLST typing. The resulting ST distribution was as follows: 52.2% (23 isolates) belonged to ST2, 40.9% (18 isolates) belonged to ST10, and the remaining 3 isolates represented distinct STs, namely ST25, ST525, and ST575 (Fig. 1).

### VF genes

Various VF genes of *A. baumannii* were grouped as shown in Table 3 into 10 different categories.

**TABLE 3:** Different virulence factors (VFs) of *A. baumannii^a^*

VF class	VFs	VF genes	Total (44)	Blood	ET and TT secretions
				No. of isolates (21)	No. of isolates (23)
Adherence	Outer membrane protein	*ompA*	43 (97.7%)	20 (45.5%)	23 (52.3%)
	LPS −0 antigen (*P. aeruginosa*) (*Pseudomonas*)		44 (100%)	21 (47.7%)	23 (52.3%)
	Type IV pili (*Neisseria*)	*pile*	44 (100%)	21 (47.7%)	23 (52.3%)
Biofilm formation	AdeFGH efflux pump/transport autoinducer	*adeF*	44 (100%)	21 (47.7%)	23 (52.3%)
		*adeG*	42 (95.5%)	20 (45.5%)	22 (50.0%)
		*adeH*	43 (97.7%)	20 (45.5%)	23 (52.3%)
	Biofilm-associated protein	*bap*	23 (52.3%)	14 (31.8%)	9 (20.5%)
	Csu pili	*csuA/B*	23 (52.3%)	15 (34.1%)	8 (18.2%)
		*csuA*	15 (34.1%)	7 (15.9%)	8 (18.2%)
		*csuB*	24 (54.5%)	15 (34.1%)	9 (20.5%)
		*csuC*	24 (54.5%)	15 (34.1%)	9 (20.5%)
		*csuD*	23 (52.3%)	14 (31.8%)	9 (20.5%)
		*csuE*	23 (52.3%)	14 (31.8%)	9 (20.5%)
	PNAG (poly-β-(1–6)-N-acetyl glucosamine)	*pgaA*	44 (100%)	21 (47.7%)	23 (52.3%)
		*pgaB*	43(97.7%)	20 (45.5%)	23 (52.3%)
		*pgaC*	43 (97.7%)	20 (45.5%)	23 (52.3%)
		*pgaD*	44 (100%)	21 (47.7%)	23 (52.3%)
Enzyme	Phospholipase C	*plc*	44 (100%)	21 (47.7%)	23 (52.3%)
	Phospholipase D	*plcD*	44 (100%)	21 (47.7%)	23 (52.3%)
Immune evasion	Capsule	Undetermined	43 (97.7%)	20 (45.5%)	23 (52.3%)
	LPS	*lpsB*	44 (100%)	21 (47.7%)	23 (52.3%)
		*lpxA*	44 (100%)	21 (47.7%)	23 (52.3%)
		*lpxB*	42 (95.5%)	20 (45.5%)	22 (50.0%)
		*lpxC*	44 (100%)	21 (47.7%)	23 (52.3%)
		*lpxD*	44 (100%)	21 (47.7%)	23 (52.3%)
		*lpxL*	44 (100%)	21 (47.7%)	23 (52.3%)
		*lpxM*	44 (100%)	21 (47.7%)	23 (52.3%)
Iron uptake	Acinetobactin	*barA*	44 (100%)	21 (47.7%)	23 (52.3%)
		*barB*	44 (100%)	21 (47.7%)	23 (52.3%)
		*basA*	44 (100%)	21 (47.7%)	23 (52.3%)
		*basB*	44 (100%)	21 (47.7%)	23 (52.3%)
		*basC*	43 (97.7%)	20 (45.5%)	23 (52.3%)
		*basD*	44 (100%)	21 (47.7%)	23 (52.3%)
		*basF*	44 (100%)	21 (47.7%)	23 (52.3%)
		*basG*	43 (97.7%)	20 (45.5%)	23 (52.3%)
		*basH*	44 (100%)	21 (47.7%)	23 (52.3%)
		*basI*	43 (97.7%)	21 (47.7%)	22 (50.0%)
		*basJ*	44 (100%)	21 (47.7%)	23 (52.3%)
		*bauA*	44 (100%)	21 (47.7%)	23 (52.3%)
		*bauB*	43 (97.7%)	20 (45.5%)	23 (52.3%)
		*bauC*	44 (100%)	21 (47.7%)	23 (52.3%)
		*bauD*	44 (100%)	21 (47.7%)	23 (52.3%)
		*bauE*	43 (97.7%)	20 (45.5%)	23 (52.3%)
		*bauF*	44 (100%)	21 (47.7%)	23 (52.3%)
		*entE*	43 (97.7%)	20 (45.5%)	23 (52.3%)
	Heme utilization	Undetermined	44 (100%)	21 (47.7%)	23 (52.3%)
		Undetermined	44 (100%)	21 (47.7%)	23 (52.3%)
		Undetermined	44 (100%)	21 (47.7%)	23 (52.3%)
		Undetermined	44 (100%)	21 (47.7%)	23 (52.3%)
		Undetermined	44 (100%)	21 (47.7%)	23 (52.3%)
		Undetermined	43 (97.7%)	20 (45.5%)	23 (52.3%)
		Undetermined	43 (97.7%)	20 (45.5%)	23 (52.3%)
		Undetermined	43 (97.7%)	20 (45.5%)	23 (52.3%)
		Undetermined	34 (77.3%)	18 (40.9%)	16 (36.4%)
		*hemO*	42 (95.5%)	20 (45.5%)	22 (50%)
Regulation	Quorum sensing	*abaI*	25 (56.8%)	16 (36.4%)	9 (20.5%)
		*abaR*	24 (54.5%)	15 (34.1%)	9 (20.5%)
	Two-component system	*bfmR*	44 (100%)	21 (47.7%)	23 (52.3%)
		*bfmS*	44 (100%)	21 (47.7%)	23 (52.3%)
Serum resistance	PbpG	*pbpG*	32 (72.7%)	15 (34.1%)	17 (38.6%)
Stress adaptation	Catalase (*Neisseria*)	*katA*	6 (13.6%)	2 (4.5%)	4 (9.1%)
Acid resistance	Urease (*Helicobacter*)	*ureG*	2 (4.5%)	1 (2.3%)	1 (2.3%)
Antiphagocytosis	Capsular polysaccharide (Vibrio)	*wbjD/wecB*	1 (2.3%)	1 (2.3%)	0
		*wecC*	1 (2.3%)	1 (2.3%)	0

^*a*^ Performed *t*-test and Mann-Whitney test using Jamovi 2.6.44.0. The *P* values were 0.113 (*t*-test) and 0.082 (Mann-Whitney), both >0.05, indicating no significant difference in VF genes between blood and respiratory isolates.

The most common VF genes found in all isolates of *A. baumannii* were *ompA* (97.7%) and *lps-o* antigen (100%) related to adherence, *adeF* (100%), *adeG* (95.5%), and *adeH* (97.7%) (AdeFGH efflux pump-related VF genes) and *pgaA* and *pgaD* (100%) related to biofilm formation, *plc* and *plcD* (100%) related to enzyme secretion and immune evasion related capsular gene (100%) and *lpsB*, *lpxA*, *lpxC*, *lpxD*, *lpxL*, and *lpxM* were found in all isolates, respectively. (Table 3)

Iron uptake-associated acinetobactin and heme utilization-related genes were *barA*, *barB*, *basA*, *basB*, *basD*, *basF*, *bauA*, *bauC*, and *bauD*, which were found in all isolates, and *basC*, *basG*, *basI*, *bauB*, and* entE* were found in 43 (97.7%) isolates, and *hemO* gene was found in 42 (95.5%) of isolates. (Table 3)

Quorum sensing-related genes *abal* and *abaR* were found in 25 (56.8%) and 24 (54.5%) of isolates, and serum resistance gene *pbpG* was found in 32 (72.7%) of isolates), whereas stress adaptation gene *katA* is present in 6 (13.6%) isolates of *A. baumannii*. (Table 3)

Table 3 shows differences in the virulence genes between *A. baumannii* isolated from blood and respiratory specimens. There was no significant difference in VF genes found in blood and respiratory *A. baumannii* isolates (*P* value > 0.5). No notable difference was observed in VF except in the *bap* gene, which was present in 14 (31.8%) blood isolates compared to 9 (20.5%) respiratory isolates. Csu pili-related VF genes were more prevalent in blood isolates than in respiratory specimens. Regarding quorum-sensing-related genes, *abaI* was present in 16 (36.4%) and *abaR* was present in 15 (34.1%) of blood isolates, whereas in respiratory isolates, *abaI* and *abaR* were each present in 9 (20.5%) isolates. Additionally, the antiphagocytosis-associated genes *wbjD* and *wecC* were found in only one strain of *A. baumannii* isolated from blood.

Table 4 shows that most of the strains of *A. baumannii* were isolated from the patients with respiratory diseases (*n* = 9), craniotomy (*n* = 9), and sepsis (*n* = 6), followed by other co-morbid conditions like chronic kidney disease, chronic liver disease, and others.

**TABLE 4:** Different clinical conditions associated with *A. baumannii* infection

Clinical condition	Total no. of patients
Post craniotomy patients	9
Diabetic + other diseases	8
Respiratory disease except tuberculosis	6
Other	6
Sepsis	4
CLD	3
Tuberculosis	3
Alcoholic	2
CKD	1
Sepsis + respiratory disease	1
Sepsis + CLD	1

**Fig 2:**
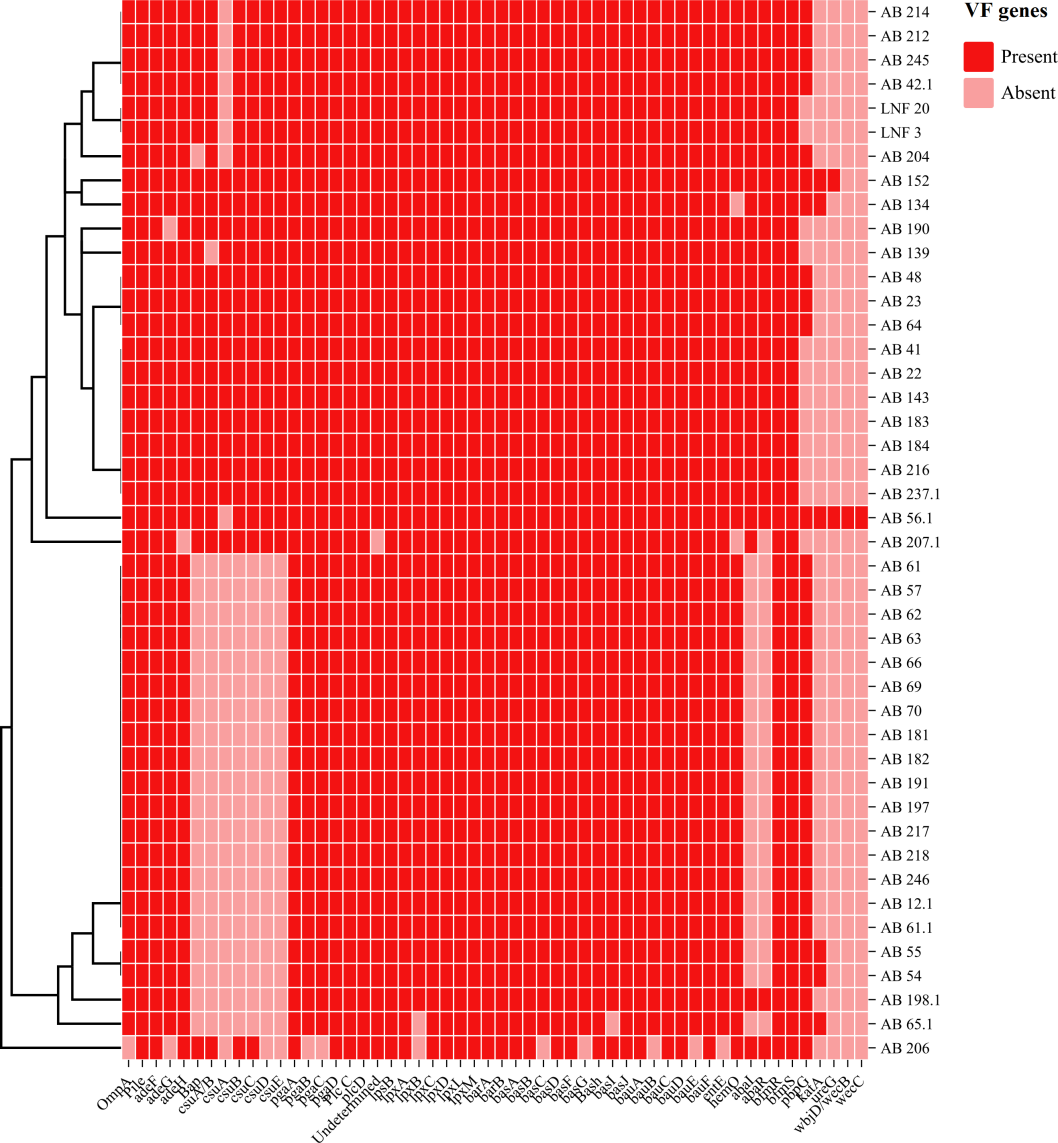
The predicted VF genes of 44 isolates of *A. baumannii*. The red-colored squares represent the presence of VF genes, and the pink-colored square represents the absence of VF genes.

**Fig 3:**
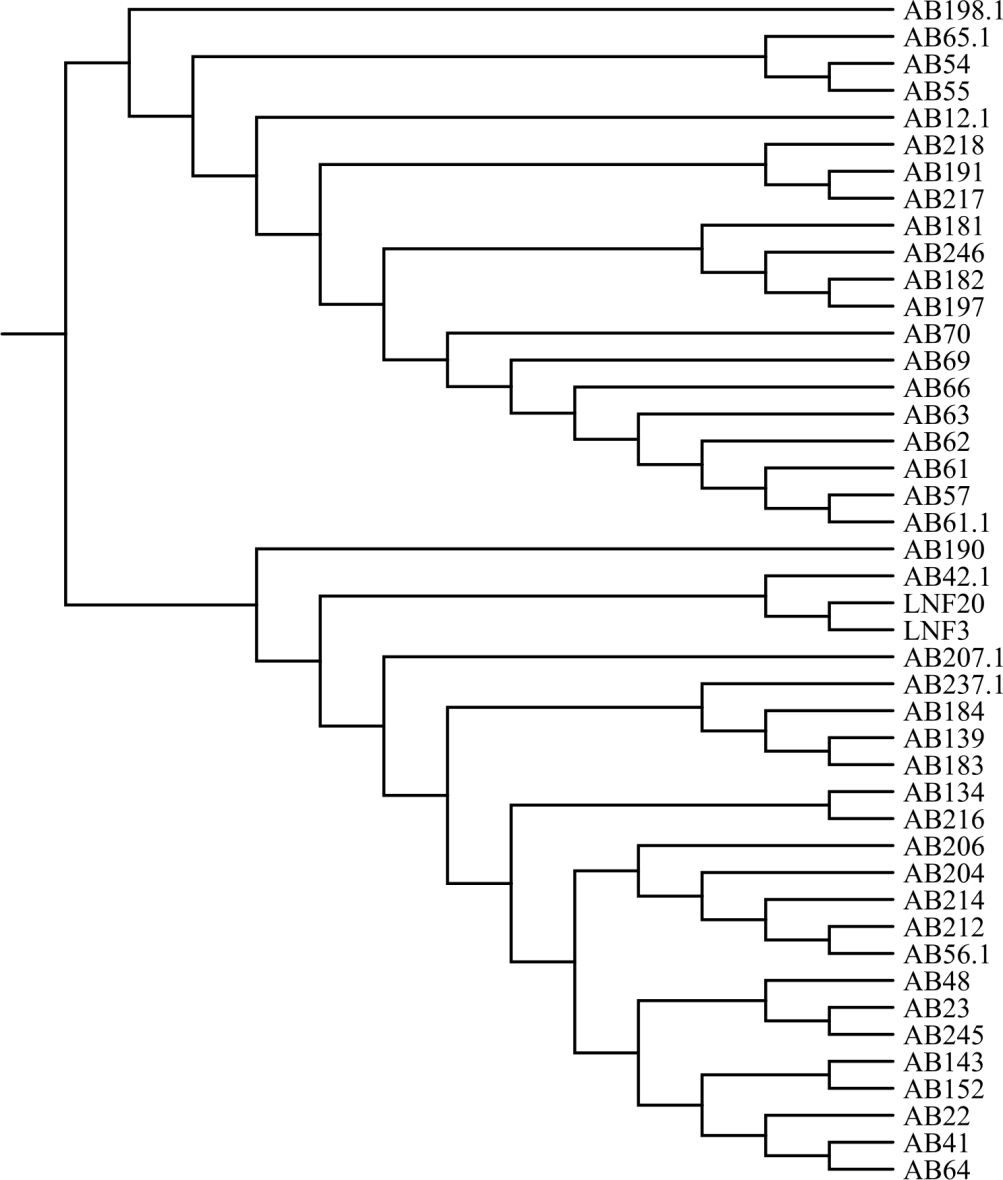
Phylogenetic tree illustrating the genetic relationships between our isolates and publicly available *A. baumannii* genomes.

Figure 2 presents a heatmap prepared by Chiplot online software illustrating the distribution of VF genes in *A. baumannii*, while Fig. 3 depicts a phylogenetic tree constructed to assess the genetic relationships between our isolates and publicly available *A. baumannii* genomes using the NDtree online tool provided by the Center for Genomic Epidemiology (17).

The overall mortality rate was reported to be around 30.69% in patients on ventilators during the study period with MDR *A. baumannii* infection.

## DISCUSSION

Hospital-acquired infections, especially VAPs, are major causes of mortality worldwide. MDR *A. baumannii*, an opportunistic pathogen, causes hospital outbreaks mainly because of genetic evolution in adaptation to hospital environment and acquisition of various VFs (12).

*A. baumannii* has evolved over the last few decades to become one of the most potent nosocomial pathogens, with an ever-increasing resistance to antibiotics (18).

Various *in vitro* and *in vivo* studies on experimental animals have added to the knowledge of virulence and pathogenesis of *A. baumannii* infections. The bacterium’s survival in the hospital environment and acquisition of many VFs during its evolution have exacerbated this issue.

In India, the majority of studies that are published are based on phenotypic and epidemiological characterization. Our study primarily focuses on the genomic analysis of VFs of MDR *A. baumannii* causing VAPs in this geographical region. We have also listed the patient’s clinical conditions and reason for the patient needing ventilator support. All the *A. baumannii* were MDR showing susceptibility to colistin and tigecycline or minocycline (19). The use of polymerase chain reaction based techniques is usually limited to the molecular characterization of one or more genetic determinants related to virulence or resistance (12, 20). WGS provides detailed genomic data of organisms that can be utilized to analyze the different VFs involved in pathogenic mechanisms by different pathogens within the hospital environment, particularly in our kind of setup (12, 18, 21).

### STs of *A. baumannii*

Our study analyzed 44 isolates using MLST according to the Pasteur Scheme and identified ST2 as the most prevalent ST, followed by ST10. The predominance of ST2, a member of the international clone II lineage, aligns with global trends, including those reported in India, highlighting its widespread dissemination (22).

### VFs of *A. baumannii*

#### Adherence and biofilm formation: outer membrane protein (OMP) and biofilm-associated protein

A critical aspect of *A. baumannii* infections is the formation of biofilms, which exhibit enhanced tolerance to antibiotics and an increased risk of developing antimicrobial resistance (7).

A study done by Shital N. Kumar predicted VFs, including adherence VFs such as biofilm-associated gene *bap*, *ompA*, poly-N-acetyl D-glucosamine (PNAG), and csu pili, which play a crucial role in biofilm formation and adherence (12). Our study revealed the presence of surface-associated VFs, including *ompA* (97.7%), biofilm-associated gene *bap* (52.3%), and PNAG-related genes *pgaA* and *pgaD* (100%).

A study by Gautam et al. detected the *ompA* gene in 281 isolates (91.53%) and the *bap* gene in 98 isolates (31.92%) (23). Our study yielded almost similar results, with the *ompA* gene and *bap* gene detected in 43 isolates (97.7%) and 23 isolates (52.3%), respectively.

Mahmoudi Monfared et al. (24) reported the prevalence of *bap*, *blaPER-1*, and *csuE* genes in *A. baumannii* isolates from various specimens as 70.3%, 54.2%, and 93.2%, respectively (24). In contrast, our study found the *bap* and *csuE* genes in 52.3% (23/44) of isolates, specifically from blood and respiratory specimens of VAP patients. The difference in findings may be due to the variation in specimen sources, highlighting the genetic variability of *A. baumannii* isolates.

Our study detected the *ompA* gene in 43 (97.7%) of mechanical ventilator-associated isolates. This finding is consistent with previous research by Thummeepak et al. (25), which reported the presence of the *ompA* gene in 169 (87.1%) out of 194 MDR *A. baumannii* isolates (25).

#### Enzyme: phospholipase C and D

The presence of phospholipase C has been linked to increased adhesion, invasion ability, and cytolytic activity of *A. baumannii* (26). Additionally, a study found that disrupting *A. baumannii* phospholipase D reduced its ability to thrive in serum, decreased its evasion of epithelial cells, and diminished its pathogenicity in a pneumonia model of mouse (27). Notably, our research revealed that nearly all *A. baumannii* strains isolated from respiratory specimens and blood possessed the *plcC* and *plcD* genes related to the phospholipase enzyme, while studies by Depka et al. and Liu et al. showed prevalence rates of the *plcD* gene as 99% and 87.5% in CRAB, respectively (18, 28).

#### Immune evasion: capsule and LPS

Capsules are necessary for evading host immune defenses, resisting antimicrobial compounds and surviving in adverse environments (29). Mucoid strains of *A. baumannii* virulence depend on capsular polysaccharide production, which is regulated by the *bfmRS* and *ompR* genes (29). In our study, we observed the *bfmRS* gene in all *A. baumannii* isolates and the *ompA* gene in 43 out of 44 isolates, while Tobin et al. also found the *bfmRS* gene in all isolates (30).

LPSs stimulate the production of inflammatory cytokines (TNF-α, IL-6, IL-1β, and IL-8) and chemokines and play an important role in the modulation of immune response (31). Colistin is used as a last-resort drug in cases of MDR *A. baumannii*, targeting LPS. Mutations in *pmrAB* genes are generally associated with LPS modification, as observed in clinical isolates of MDR *A. baumannii* (32).

In our study, capsular genes were detected in 43 (97.7%) of the *A. baumannii* isolates, and the LPS genes *lpxA*, *lpxC*, and *lpxD* were detected in all isolates, except for *lpxB*, which was detected in 42 (95.5%) isolates. In contrast, a study by Kamoshida G on colistin-resistant mutants of *A. baumannii* found that *lpxA*, *lpxC*, and *lpxD* genes were detected in 21, 36, and 14 colistin-resistant strains, respectively (32).

#### Iron uptake: acinetobactin and heme utilization

In a study conducted by Liu et al. (33), it was found that environments rich in iron increase the expression of the OmpA protein in *A. baumannii* (33). Strains with higher OmpA expression were observed to be more invasive, potentially contributing to *A. baumannii* infection and pathogenicity (33). Consistent with these findings, our study detected the *ompA* gene in 97.7% (43/44) of isolates. Additionally, iron uptake-related genes, such as acinetobactin and heme utilization genes, were present in 95.5% of isolates, emphasizing the significance of iron acquisition in *A. baumannii* pathogenesis.

In a study conducted by Artuso et al. (34), it was discovered that 67% (*n* = 717) of *A. baumannii* isolates possess siderophore clusters (34). Notably, 98% (*n* = 1,053) of the isolates carried both baumannoferrin (bfn) and acinetobactin (bas/bau) siderophore clusters, while 68% (*n* = 728) carried both *hemO* and *hemT* haem-uptake clusters (34). Our study aligns with these findings, as we identified acinetobactin (*bas/bau*) in approximately 97.7% (43/44) of the isolates, and the heme uptake cluster *hemO* in 95.5% (42/44) of the isolates.

#### Regulation: quorum sensing and two-component system

Sun et al. used the *Galleria mellonella* model to study a quorum sensing system, *abaI/abaR* role in *A. baumannii* ATCC 17978 virulence. They found that strains without *abaI* were less virulent, while *abaR* mutants were more pathogenic (35). In our study, we detected the *abaI* gene in 56.8% and the *abaR* gene in 54.5% of *A. baumannii* isolates recovered from sepsis patients on ventilators.

Two-component system, bfmRS, is modulated by the expression of the csu operon (36). Notably, inactivation of the *bfmR* and *bfmS* genes has been demonstrated to decrease the formation of biofilm in XDR *A. baumannii* isolates (37, 38). Consistent with these findings, Law et al. reported that disruption of the *csu* genes in *A. baumannii* significantly impairs biofilm formation (11).

#### Serum resistance: *PbpG*

Our analysis revealed that all isolates harbored the *PbpG* gene, which is essential for bacterial cell wall stability and resistance to human serum (39).

### Stress adaptation

*A. baumannii* strains have evolved to counter oxidative stress by acquiring ISAba1 and the *katG* gene, which encodes the catalase enzyme. The *katG* gene enhances resistance to hydrogen peroxide (40). Our study analyzed 44 isolates and found that 6 (13.6%) carried the *katA* gene.

Many studies on the prevalence and mortality of *A. baumannii* infections causing HAP and VAP have reported varying rates, with prevalence ranging from 55% to 100% and mortality rates ranging from 28% to 68%, across different regions and countries (41).

These differences may be attributed to variations in medical practices and facilities (41). Studies have reported varying mortality rates due to CRAB, with Russo et al. reporting 65.4% (42) and Lodise et al. reporting 20.5% (43). In our study, the mortality rate attributed to MDR *A. baumannii* was 30.69%.

No significant differences in VF genes were observed in isolates of blood and respiratory specimens.

The key VFs identified in this study were associated with various functions, including biofilm formation and adherence such as *ompA, lps-o*, pili, adeFGH efflux pump, and PNAG genes, enzymatic activity like *plcD*, immune evasion through the capsule and lps genes, iron acquisition via acinetobactin and heme utilization genes, and two-component signaling systems involving *bfmR* and *bfmS* genes.

These findings will facilitate future opportunities for the development of novel strategies to combat *A. baumannii*. For example, *A. baumannii* strains exhibiting phenotypic carbapenemase resistance due to efflux pumps, rather than carbapenemase enzyme production, may be effectively treated using chemicals such as *Schoepfia schreberi* bark extracts, which modulate efflux pump function and render carbapenems effective (44). Similarly, agents can be developed to target biofilm formation, iron chelation, and immune modulation to block LPS-induced cytokine storms.

### Conclusion

This is the first study about the genetic analysis of different VFs of *A. baumannii* done by WGS in Indian ICU setup. These findings have improved our understanding of the various VFs responsible for the survival of *A. baumannii* in the hospital environment, their role in pathogenic mechanisms, and the success of this pathogen as a major nosocomial threat. This study could be further utilized to bring down the rate of nosocomial infections by this pathogen by targeting its VFs by modifying the hospital infection control measures. The study also suggests future strategies to focus on developing approaches to target OMP products and inhibit secretions by outer membrane vesicles, tailored to specific OMP types related to a particular geographical location.

## Data Availability

All *A. baumannii* isolates genome data have been uploaded to the NCBI repository under Bioproject accession number PRJNA1234895.
